# UNDERLYING CAUSES OF DEMENTIA-RELATED ANXIETY AMONG COGNITIVELY-HEALTHY INDIVIDUALS

**DOI:** 10.1093/geroni/igad104.1961

**Published:** 2023-12-21

**Authors:** Molly Maxfield, Allie Peckham, Dara James

**Affiliations:** Arizona State University, Phoenix, Arizona, United States; Arizona State University, Phoenix, Arizona, United States; Arizona State University, Tempe, Arizona, United States

## Abstract

Alzheimer’s disease and related dementias (ADRD) are prevalent, without cures, and contribute to declining functional independence. Their widespread effects make managing ADRD financially, socially, and psychologically difficult, and present challenges for the diagnosed individual as well as their family, friends, and caregivers. These features represent at least some of the reasons ADRDs are feared diagnoses. Dementia-related anxiety (DRA) is anxiety about a current or potential ADRD diagnosis. In a mixed methods study designed to identify causes of DRA, we used semi-structured interviews to assess participants’ thoughts, feelings, and reactions regarding ADRD. Participants included 50 cognitively healthy adults aged 58 to 89 (M = 70.80, SD = 6.02; 64% female). Interviews were analyzed using an inductive iterative thematic approach. All transcripts were coded by two researchers. Relying on this iterative process, we adopted and revised themes as more responses and codes were reviewed. The thematic analysis revealed participants described how the progression of symptoms would destabilize their independence, thereby undermining their identity and relationships in ways that were anxiety provoking. Participants also noted various coping strategies that help manage DRA: making care plans for the future, engaging in healthy, feeling supported by family and friends, and accepting that disappointments, including ADRD diagnoses, are simply a part of life. Results offer insight into factors that contribute to and help reduce DRA, which can be used to inform public health messaging and develop applicable and clinically relevant interventions to meet the needs of individuals experiencing DRA.

